# The Role of Conspiracy Theories in the Spread of COVID-19 across the United States

**DOI:** 10.3390/ijerph18073843

**Published:** 2021-04-06

**Authors:** Fu Gu, Yingwen Wu, Xinyu Hu, Jianfeng Guo, Xiaohan Yang, Xinze Zhao

**Affiliations:** 1 Center of Engineering Management, Polytechnic Institute, Zhejiang University, Hangzhou 310027, China; gufu@zju.edu.cn; 2 Department of Industrial and System Engineering, Zhejiang University, Hangzhou 310027, China; 11825089@zju.edu.cn (Y.W.); 98huxinyu@zju.edu.cn (X.H.); 3 National Institute of Innovation Management, Zhejiang University, Hangzhou 310027, China; 4 Institute of Science and Development, Chinese Academy of Sciences, Beijing 100190, China; yangxiaohan20@mails.ucas.ac.cn (X.Y.); 2017011746@student.cup.edu.cn (X.Z.); 5 School of Public Policy and Management, University of Chinese Academy of Sciences, Beijing 100049, China

**Keywords:** COVID-19, conspiracy theory, human mobility, mediation analysis, official information, personal protection

## Abstract

The outbreak of coronavirus disease 2019 (COVID-19) inspires various conspiracy theories, which could divert public attention, alter human behaviors, and consequently affect the spread of the pandemic. Here we estimate the relation of the online attention on COVID-19-related conspiracy theories to human mobility, as well as to the numbers of confirmed COVID-19 cases, during 14 March 2020 to 28 August 2020. We observe that the online attention to COVID-19 conspiracy theories is significantly and negatively related to human mobility, but its negative impact is noticeably less than those of the attention to official information and personal protection measures. Since human mobility significantly promotes the spread of COVID-19, the attention to official information and personal protection measures lowers COVID-19 cases by 16.16% and 9.41%, respectively, while attention to conspiracy theories only reduces the COVID-19 cases by 6.65%. In addition, we find that in the states with higher online attention to COVID-19 conspiracy theories, the negative relation of the attention to conspiracy theories is much weaker than that in states where there is less concern about conspiracies. This study stresses the necessity of restricting the online transmission of unfounded conspiracy theories during a pandemic.

## 1. Introduction

During an epidemic, misinformation proliferates as fear grows, posing further damage to public health. Coronavirus disease 2019 (COVID-19) is also a global misinformation pandemic; conspiracy theories in various forms, such as fifth-generation (5G) technology causes COVID-19 and the Deep State masterminded the pandemic, are spreading along with the virus [1,2,3]. A number of people firmly believe one influential conspiracy theory: COVID-19 does not indeed exist, and the pandemic is a plot by global elites to rule the world [3]. They refused to wear masks [4] and launched protests against quarantine [5]. Facilitated by the Internet, these conspiracy theories have proliferated as the pandemic has worsened. Billions of users have been exposed to unfounded conspiracy theories, false statistics and other rumors, though social networks have taken measures to contain the spread of COVID-19 related misinformation [6,7]. Despite personal belief in conspiracy theories related to self-selection and predispositions [8,9], the spread of such misinformation could still divert public attention and subsequently affect the behaviors of a large number of people. It is of critical importance to understand the potential linkage between the attention on COVID-19 conspiracy theories, human behaviors and virus transmission. Focusing on the United States, in this article we empirically estimate the relation of the online attention to COVID-19-related conspiracy theories to human mobility, which is proved to be a primary driver of COVID-19 transmission [10,11,12,13].

We construct a daily panel data set that includes all state-level administrative regions in the United States, that is, 50 states and 1 district, spanning from 14 March 2020 to 28 August 2020. Before 14 March 2020, most of the COVID-19 conspiracy theories had not been posted on the Internet, as there were only a few hundred COVID-19 cases reported back then. In the beginning of September 2020, public attention turned to the 2020 presidential election, as mail-in ballots had started at the end of August 2020. Our dataset contains the online attention to various COVID-10-related conspiracy theories, the aggregate human mobility data collected from the Google’s COVID-19 Community Mobility Report (CCMR, source: https://www.google.com/covid19/mobility/, accessed on 1 December 2020), the number of the reported COVID-19 cases that is provided by Johns Hopkins University (JHU, source: https://github.com/CSSEGISandData/COVID-19, accessed on 1 December 2020), and the online attention on official information (e.g., *Reopen*, *Homeschooling*, *School Closure*) and personal protection measures (e.g., *Social distancing*, *Face mask*, *Handwashing*). Except for the number of the reported COVID-19 cases, all the above data series are standardized within the range of (0, 1). We employ a mediation analysis (or sometimes referred to as *casual step analysis*) [14,15] to conduct our empirical estimation, in which mobility is considered as a mediator between the online attention series and the number of the COVID-19 cases. The methodology is extensively used in identifying potential casual relations [16,17,18,19]. The complete lists and selection process of keywords, and our empirical models are elaborated in the following section—Section 2.

Notably, we only consider the online attention series due to the following three reasons. First, the Internet plays a noticeably important role in the spread of the COVID-19 conspiracy theories during the pandemic [1,2], although other channels such as phone calls and television shows are still conveying misinformation. Second, selective exposure is the primary driver of online misinformation transmission [20]; this feature is compatible with the role of self-selection and predispositions in the belief of conspiracy theories [8,9]. Third, online information propagation can be quantified based on the service provided by public search engines such as Google, while the information about other means of misinformation transmission such as phone calls and text messages cannot be easily acquired. Following the previous literature [21,22,23,24,25,26,27], we use the search intensities of the keywords related to COVID-19 conspiracy theories, official information and personal protection measures (provided by Google Trends) to denote the online attention on these particular issues. In essence, search keywords and their search intensities are corresponding to the two fundamental components of the classical attention theory [28], namely attention selection and attention intensity.

## 2. Materials and Methods

### 2.1. Selection of Keywords

From the literature that examines COVID-19 conspiracy theories [1,2,3,29] and online contents such as Tweets, blogs, columns and posts, we initially include over 40 widely spread COVID-19 conspiracy theories, see Table A1 in the Appendix A. Afterwards, we try different combinations of keywords that denote these conspiracy theories on Google Trends, and compare these keywords with the hottest topics on Google in the United States. Hence, based on the values of the search intensities derived from the trials, we select 16 most representative conspiracy theory keywords, namely *Biological Weapon*, *Coronavirus Patent*, *Chinese Lab*, *Wuhan Institute of Virology*, *Bill Gates*, *George Soros*, *Deep State*, *Population Control*, *Microchip*, *Vaccine Conspiracy*, *5G Conspiracy*, *Hydroxychloroquine*, *Bleach*, *Colloidal Silver*, *COVID Party*, and *QAnon*. Here we briefly explain our selection as follows. Conspiracy theorists claim that COVID-19 is a *Biological Weapon* or a *Coronavirus Patent*, escaped from some *Chinese Lab* or more specifically the *Wuhan Institute of Virology*; such myths have been repeatedly denied by scientific communities [30,31]. The COVID-19 pandemic is masterminded by *Bill Gates*, *George Soros* or the *Deep State* of the United States (i.e., a group of elites that controls the country and manipulates the United States government), to enforce *Population Control* or to implant *Microchip* to control the general public via *Vaccine Conspiracy*. The COVID-19 outbreak is related to the use of fifth-generation (5G) technologies; this is *5G Conspiracy*. The miracle cures to COVID-19 are *Hydroxychloroquine*, *Bleach* and *Colloidal Silver*, but their usage is arrested by the United States government [32,33]. In fact, hydroxychloroquine has yet not been proved to effective in treating COVID-19 [34,35]. *COVID Party* originally refers to someone who did not believe the existence of the virus that died from COVID-19 after attending a party, but the story has many versions [36,37] and sometimes has been linked to religion [38]. *QAnon* is a group of conspiracy theorists who actively compose COVID-19 misinformation [39,40], and this keyword is selected due to its frequent coappearances with the other COVID-19 conspiracy theory keywords.

To denote online attention to official information, we choose the following keywords: *National Quarantine*, *Reopen*, *Homeschooling*, *School Closure*, *Anthony Fauci*, *Unemployment*, *Mortgage Forbearance*, and *Trump Approval*, according to their relevance, significance and representativeness. The first four keywords are related to different governmental interventions, which have been proved to be effective in containing this epidemic [10,12,41,42]. Dr *Anthony Fauci* is the Director of the National Institute of Allergy and Infectious Diseases and is considered as a leader in the fight against the COVID-19 pandemic. The outbreak creates a global economic crisis, referred to as the Great Lockdown [43], therefore *Unemployment* and *Mortgage Forbearance* are included. *Trump Approval* is considered due to his controversial responses to the pandemic, for examples, suggesting the injection of disinfectants to cure COVID-19 [33], and making unexpected comments on the development of COVID-19 [44].

We select the following keywords to denote personal protection measures: *Face Mask*, *Surgical Mask*, *Handwashing*, *Sanitizer*, *Social Distancing*, and *COVID Test*. Similarly, the selection is based on the keywords’ relevance, significance and representativeness. *Face Mask*, *Surgical Mask*, *Handwashing*, *Sanitizer*, and *Social Distancing* can effectively reduce the likelihood of being infected by COVID-19, and voluntarily taking a *COVID-19 test* is also considered as a self-protection measure.

Using the event study method proposed by Ji and Guo [22], we ensure that the online attention series of these keywords are generally synchronous with the outbreak of COVID-19 in the United States in our selected study period, i.e., 14 March 2020 to 28 August 2020. Adopting the online attention processing approach of Da et al. [21], we aggregate the search intensities of all the aforementioned keywords in each state or district from *Google Trends*, representing online attention on the includes issues.

**Figure 1:**
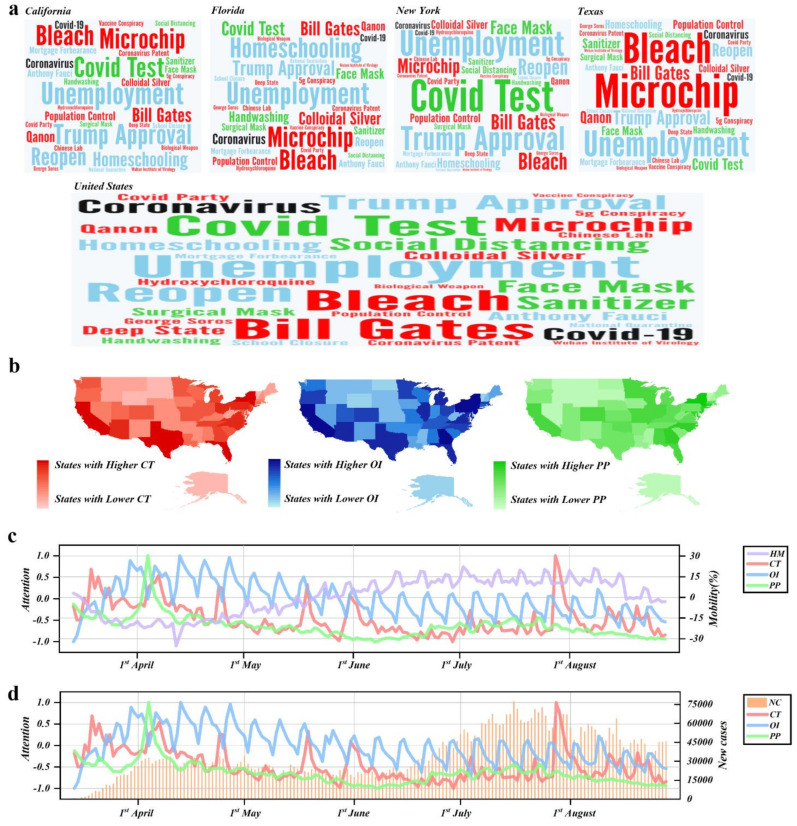
(**a**) The keyword clouds of the top search queries on *Google* in California, Florida, New York and Texas, as well as in the whole country. (**b**) The heat maps of the United States, based on the online attention on the selected keywords of the conspiracy theories and official information. (**c**) The co-movements between the summed human mobility, and the online attention to the keywords related to the coronavirus disease 2019 (COVID-19) conspiracy theories, official information and personal protection measures. (**d**) The co-movements between the reported COVID-19 cases and the online attention on the keywords related to COVID-19 conspiracy theories as well as to official information and personal protection measures. *NC* denotes the number of the reported COVID-19 cases, *HM* denotes the aggregate human mobility, *CT* denotes the combined online attention on the selected keywords that are related to COVID-19 conspiracy theories, *OI* denotes the combined online attention to the selected keywords that are related to official information, and *PP* denotes the combined online attention to the selected keywords that are related to personal protection measures.

Figure 1a shows the keyword clouds of the top search queries on *Google* in populated states such as California, Florida, New York and Texas, as well as those at the national level. The sizes of the included keywords confirm the validity of our selections. Figure 1b presents the heat maps that are based on online attention to the keywords that represent the conspiracy theories, official information and personal protection measures. Based on the national average value of the conspiracy theory attention series during the studied period, we sort all the states into two categories, namely the states with higher online attention to conspiracy theories and the states with lower online attention on conspiracy theories, as shown in Table A2 in the Appendix A. Figure 1c,d imply that there may exist some potential linkages between the numbers of the reported COVID-19 cases, human mobility and the three types of the online attention series. Hence, further empirical estimation is required to quantify the potential linkages.

### 2.2. Construction of Empirics

We employ a fixed effect framework, which accounts for unobservable state-invariant heterogeneity [45,46], to empirically test the potential linkage between the online attention series, the human mobility and the number of the reported COVID-19 cases. We begin by using the following five models:

(1) $$NC_{it+7}=\alpha_{1}+\beta_{1}HM+u_{i}+\varepsilon_{it}$$

(2) $$NC_{it+7}=\alpha_{2}+\beta_{2}CT+u_{i}+\varepsilon_{it}$$

(3) $$HM_{it}=\alpha_{3}+\beta_{3}CT+u_{i}+\varepsilon_{it}$$

(4) $$NC_{it+7}=\alpha_{4}+\beta_{4}CT+\beta_{5}HM+u_{i}+\varepsilon_{it}$$

(5) $$HM_{it}=\alpha_{5}+\beta_{6}OI+u_{i}+\varepsilon_{it}$$

(6) $$HM_{it}=\alpha_{6}+\beta_{7}PP+u_{i}+\varepsilon_{it}$$

Model I tests the relation of *HM* to *NC*, Model II, III and IV examine the mediating effect of *HM* in-between *CT* and *NC*, and Model V and VI test the relation of *OI* and *PP* to *HM*, respectively. The configuration of Model II, III and IV fits the definition of mediation analysis [14,15]. Since 75% of all COVID-19 patients become symptomatic within 6 or 7 days [47], an incubation period of 7 days is set in Model II and IV.

The percentages of the COVID-19 cases related to the three online attention series can be calculated as follows:

(7) $${\mathrm{Percentage}}_{CT}=\frac{\bar{CT}\;\times \beta_{3}\times \beta_{1}}{\bar{NC}}$$

(8) $${\mathrm{Percentage}}_{OI}=\frac{\bar{OI}\;\times \beta_{6}\times \beta_{1}}{\bar{NC}}$$

(9) $${\mathrm{Percentage}}_{PP}=\frac{\bar{PP}\;\times \beta_{7}\times \beta_{1}}{\bar{NC}}$$

In Model I, the Durbin–Wu–Hausman test rejects the null hypothesis, indicating that *HM* is not sufficiently exogenous to *NC*. To eliminate the influence of possible endogeneity, we employ an instrumental variable estimation with two stage least square estimator [48,49]. In this case, we choose the daily price of Bitcoin as an instrumental variable that estimates *HM*, to account for the potential endogeneity. The Cragg–Donald Wald F statistic of the Bitcoin daily price is 2691.415, suggesting that the selected instrumental variable has sufficient explanatory power for the endogenous regressor. Therefore, the selection of the instrumental variable is valid.

## 3. Results

**Figure 2:**
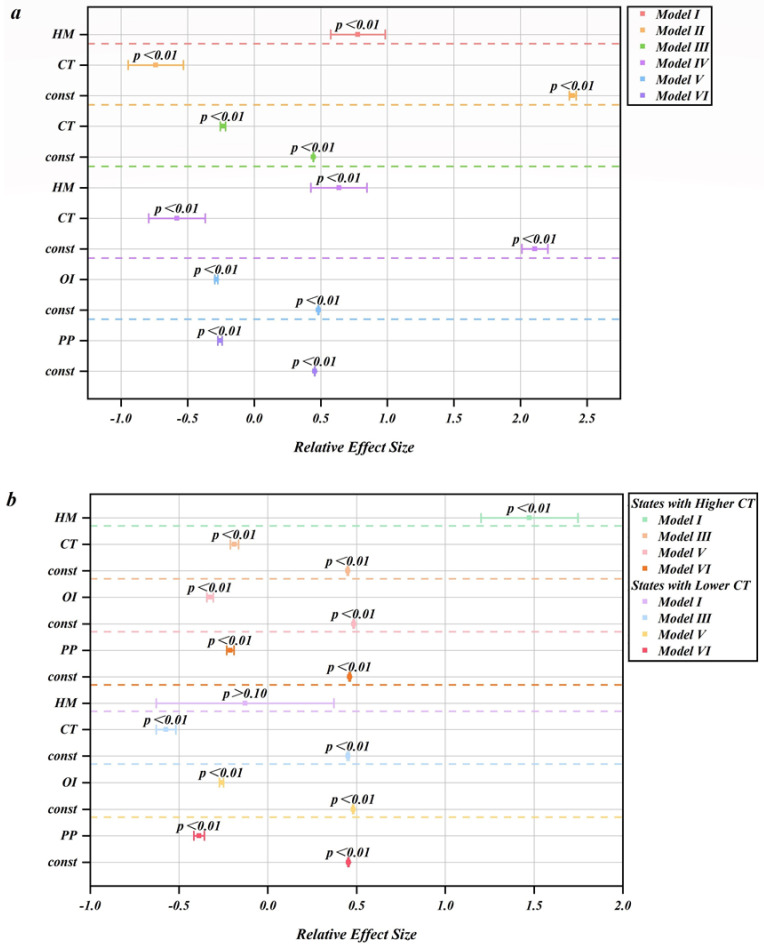
(**a**) Based on the national level dataset, line 1 to 6 display the empirical results of our mediation analysis, i.e., Models I to VI. (**b**) Displays the results of the empirical estimation (Models III, I, V and VI) using the dataset of the classified states (see Table A2).

The empirical results of all the states and the categorized states are plotted in Figure 2a,b, respectively. In general, from Figure 2a, we confirm that the aggregate human mobility is positively correlated to the numbers of the daily confirmed COVID-19 cases (*p* < 0.01), proving that human mobility can be a driver of COVID-19 transmission.

### 3.1. All the States

We find that the values of all the three online attention series are significantly and negatively related to human mobility (*p* < 0.01). This observation indicates that the online attention to the three types of content related to COVID-19 would prevent the general public from travelling as much as they usually do. For believers of the COVID-19 conspiracy theories, the misinformation could propagate the fear of this pandemic and thereby affect their traveling behaviors, even though such stories have no factual basis. For non-believers of conspiracy theories, accessing such online contents would not affect their behaviors; the reduction in their mobility may be attributed to their compliance with governmental regulations as well as the fear of being infected by COVID-19. The other two types of information delivers the gravity of the pandemic and the significance of self-protection. In this sense, increased online attention to such information goes hand in hand with the reduced human mobility.

Combining the results derived from Models II, III and IV in Figure 2a, it suggests that all three types of online attention series could reduce the COVID-19 transmission through reducing the human mobility. Yet again, amongst the three online attention series, the attention on the COVID-19 conspiracy theories exhibits the least preventive effect on the human mobility as well as on the number of the reported COVID-19 cases; this attention series can only reduce cases by 6.65%, while the attention on the official information and the personal protection measures can reduce the confirmed COVID-19 cases by 16.16% and 9.41%, respectively. The finding implies that as such misinformation proliferates on the Internet, its impact can be highly differentiated due to its different forms and contents, even for conspiracy theorists. For example, a fraction of the conspiracy theory believers keep on believing that COVID-19 is a plot that never actually took place [3], thus they did not alter their travelling patterns and even gathered around to boycott stay-at-home orders [5]. Consequently, these conspiracy theorists could even contribute more mobility than they usually did. However, the other conspiracy theorists might be afraid of the undesirable outcomes of COVID-19, as the virus is rumored to be an effective biological weapon to control the population [1,2,3]. For the non-believers, searching the stories on the Internet is purely out of their curiosity, and they would still follow official instructions to enforce social distancing and home schooling. By contrast; the functional route of online attention to the official information and personal protection measures on the COVID-19 cases is relatively simple in nature, as such information delivers a consistent message, that is, COVID-19 is an infectious disease that shall be contained.

### 3.2. Categorized States

As shown in the first and second lines of Figure 2b, we find that in the states with the higher online attention values on the COVID-19 conspiracy theories, the negative impact of the online attention on the conspiracy theories on the human mobility is noticeably weaker than that of the national level (see Figure 2a). This observation implies that in such regions, the unfounded stories that deny COVID-19 may find more believers, resulting in higher attention to conspiracy theories and less reduction in human mobility. From the third and fourth lines of Figure 2b, we find that the negative relation of the online attention on the COVID-19 related conspiracy theories to the human mobility weaker than those on official information and personal protection measures, showing a good consistency with the observation of the national level data.

However, as suggested by the third and fourth lines of Figure 2b, in states with lower online attention to the COVID-19 conspiracy theories, the preventive effect of such attention to the human mobility is much stronger than that of the national level (see Figure 2a). The observation can be explained that in these states, people occasionally googled the conspiracy keywords for the sake of satisfying their curiosity or concerning the development of this epidemic. By contrast with the states with the higher online attention to the conspiracy theories, there is little doubt about the existence or consequences of this disease among the public in these states. Therefore, in such regions, human mobility is noticeably decreased with increasing online attention to COVID-19 conspiracy theories. Besides, from the seventh and eighth lines of Figure 2b, we find that the negative relation of the online attention to conspiracy theories to the human mobility is greater than those of the official information and personal protection measures. This finding suggests that in these states, the spread of the conspiracy theories can be related to fear rather than denial of the occurrence of COVID-19, and thereby reducing the human mobility to a greater extent.

### 3.3. Instrumental Variable Analysis

**Figure 3:**
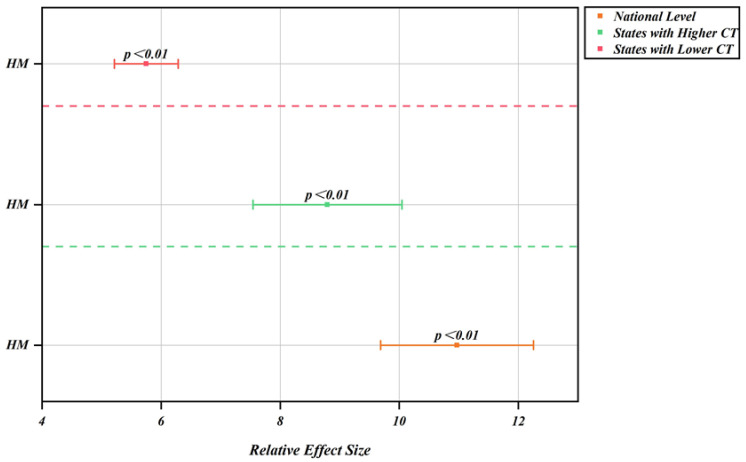
Displays the empirical results of our instrumental variable analysis, i.e., Model I with human mobility (*HM)* replaced by an estimator that is based on the daily Bitcoin price, using the dataset of the national level as well as the classified states, see Table A2.

The results of our instrumental variable analysis are shown in Figure 3, and they confirm that the human mobility has a positive relation to the numbers of COVID-19 cases. The finding fits the observations from the extant studies [10,11,12,13]; human mobility is positively correlated to the transmission of COVID-19, and travel restriction can be the most effective measure in containing this pandemic [10,12,41,42].

## 4. Discussion

Our research pioneers to empirically investigate the relation of online attention to COVID-19-related misinformation, in this case, COVID-19 conspiracy theories, to the aggregate human mobility and the number of reported COVID-19 cases in the United States. Although the types, sources and undesirable consequences of the COVID-19-related misinformation have been portrayed [1,2,29], how is such misinformation related to the number of the reported COVID-19 cases still remains unexplored before this work. Without an in-depth understanding of the potential functional route of COVID-19 misinformation on the spread of the pandemic, administrators may not be able to propose or implement any effective measures to minimize the negative outcomes of the rumors on the Internet, though they have already taken actions against such misinformation [6,7].

In sum, empirical observations show that the spread of the epidemic-related misinformation likes the epidemic itself, which has some distinctive regional characteristics. Notably, the relation of human mobility to the number of the reported COVID-19 cases remains positive and significant in the states with the higher online attention to conspiracy theories (and also confirmed by the results of our instrumental variable analysis, see Figure 3), while such a relationship becomes insignificant in the other states (see Figure 2b). Clearly, these findings confirm the presence of regional differences in the relation of online misinformation to the spread of COVID-19. The finding also agrees with the previous literature [50]. The observed regional differences could possibly be explained by the psychological basis for adopting or rejecting conspiracy theories, that is, self-selection and predispositions [8].

This study stresses the importance of containing the online spread of misinformation during an epidemic, for misinformation such as conspiracy theories not only affect public perception [51], but also could influence human behaviors. In particular, our analysis highlights the importance of suiting rumor-containing measures to local conditions, as suggested by the observed regional differences. In regions with higher attention to unfounded conspiracy theories, proliferation is related to the compromises of epidemic prevention, therefore more rigorous censorship on online content can be implemented. However, in regions with lower attention on such misinformation, there is little necessity to enforce strict rumor control measures. After all, our work shows that it is of critical importance to monitor the levels of online attention on misinformation, because this data can be an insightful reference for policy making.

One of the major limitations of this work lies in the lack of individual-level behavioral data; without such data, we can neither trace the behaviors of rumor believers and non-believers, nor their health conditions. The other primary limitation of our research is the sole focus on online attention. In fact, rumors also spread via other channels such as phone calls, text messages and television shows. Our future research would seek more comprehensive individual behavioral data, which is consistently in short supply, in order to examine the functional routes of misinformation on the epidemic transmission.

## 5. Conclusions

As the COVID-19-related conspiracy theories proliferate on the Internet [1,2,3], it is crucial we estimate the relation of the online attention on such misinformation to human behaviors, in this case, mobility, as well as to the spread of the pandemic. Our empirical findings indicate that in the United States, the negative impact of the online attention to COVID-19-related conspiracy theories on human mobility is much smaller than those of the online attention to official information and personal protection measures, suggesting that the attention and belief on this misinformation could affect human behaviors, in this case, human mobility. Such a relation shows some degree of regional difference; the preventive effect of the online attention to COVID-19 related conspiracy theories to the human mobility is much weaker in states with the higher online attention to conspiracy theories.

## Data Availability

The data presented in this study are available on request from the corresponding author.

## Appendix A

**Table A1:** Popular coronavirus disease 2019 (COVID-19) conspiracy theories on the Internet.

Summary of the Conspiracy Theory	Link
There’s no question that the someone in the government well as some websites have weaponized information about the origins of the virus. President Trump has referred to the virus as the “Chinese virus” and the “Wuhan virus”, which has aroused the suspicion about the origin.	https://www.cjr.org/the_media_today/china_coronavirus_wuhan_lab_trump.php (accessed on 1 December 2020)
At the Lincoln Memorial in Washington DC, Trump said he thought China made a horrible mistake and they did not want to admit it. He implied that China acted in some ways that ensured the virus’s spread.	https://thewire.in/world/the-coronavirus-conspiracy-theory-doesnt-add-up-its-also-a-self-defeating-diversion (accessed on 1 December 2020)
A few conservatives peddle claimed the virus did not emerge in the Wuhan market where most experts believed it had appeared, but at the Wuhan Institute of Virology. In late January, The Washington Times suggested that scientists at the lab had developed the disease as part of China’s biowarfare program.	https://newrepublic.com/article/158101/conservatives-believe-chinese-lab-created-coronavirus (accessed on 1 December 2020)
Several groups and individuals are circulating false rumors claiming that virus has been patented. Someone proclaims that the virus was created in a lab and patented in 2015.	https://www.usatoday.com/story/news/nation/2020/01/25/wuhan-coronavirus-bogus-conspiracy-theory-spreads-social-media/4569180002/ (accessed on 1 December 2020)
Institute Pasteur, the Centre National de la Recherche Scientifique, and the Université Paris VII were accused of holding coronavirus patent by a video. They might have created the COVID-19 coronavirus and released it in Wuhan.	https://www.mcgill.ca/oss/article/covid-19-pseudoscience/patently-false-disinformation-over-coronavirus-patents (accessed on 1 December 2020)
About one in four Americans think the idea that the virus was engineered in a Wuhan laboratory is a “reliable” claim. The more they believe in conspiracy theories, the lower their willingness to get vaccinated.	https://www.businessinsider.com/one-in-four-americans-believe-coronavirus-engineered-wuhan-lab-2020-10 (accessed on 1 December 2020)
Orchestrated by “big pharma” companies in conjunction with Bill Gates, the scheme would supposedly “kill millions” in the name of generating profit.	https://theconversation.com/coronavirus-and-conspiracies-how-the-far-right-is-exploiting-the-pandemic-145968 (accessed on 1 December 2020)
On 19 March, the website Biohackinfo.com falsely claimed that Gates planned to use a coronavirus vaccine as a ploy to monitor people through an injected microchip or quantum-dot spy software. Two days later, traffic started flowing to a YouTube video on the idea. It’s been viewed nearly two million times	https://www.nature.com/articles/d41586-020-01452-z (accessed on 1 December 2020)
The goal of the COVID-19 pandemic is to implant microchips in people. There is the belief that the COVID-19 tests involve a swab that reaches “to the brain”, or, with a wand which is inserted deep into the brain and injures the blood-brain barrier, as a result creating an “entry point” for infections. Besides, it is also thought that masks contain a 5G chip.	https://www.ekathimerini.com/256809/opiopin/ekathimerini/comment/conspiracy-thetheor-interests-and-microchips (accessed on 1 December 2020)
Microsoft founder Bill Gates is attempting to implant microchips into billions of people through a coronavirus vaccine, and that Dr. Anthony Fauci said every American should be microchipped.	https://www.forbes.com/sites/andrewsolender/2020/07/08/they-want-to-put-chips-inside-us-kanye-west-cites-debunked-anti-vaccine-conspiracy-theories/?sh=295754c024b8 (accessed on 1 December 2020)
The conspiracy theorists claimed that the patent, an insertable microchip system which is applied for the patent earlier this year by Microsoft Technology, is related with vaccine conspiracy.	https://www.daily-chronicle.com/2020/11/19/guest-column-the-latest-in-covid-19-conspiracy-theories-involves-the-new-vaccines/acej32t/ (accessed on 1 December 2020)
Bill Gates and George Soros want to secretly stick a chip in you while testing you for the coronavirus. What’s more, there are illustrations show how to insert the microchip with a long swab into the person’s nasal passage.	https://www.politifact.com/factchecks/2020/may/28/facebook-posts/theres-no-plot-microchip-people-during-covid-19-te/ (accessed on 1 December 2020)
George Soros owns the WuXi Pharma Lab located in Wuhan, China where COVID-19 was developed and conveniently broke out.	https://www.politifact.com/factchecks/2020/mar/19/facebook-posts/conspiracy-theory-falsely-connects-george-soros-co/ (accessed on 1 December 2020)
The coronavirus pandemic is thought to be a cover for a plan to implant trackable microchips and that the Microsoft co-founder Bill Gates is behind it.	https://www.bbc.com/news/52847648 (accessed on 1 December 2020)
Research groups funded by Bill Gates engineered and patented the novel coronavirus and will profit from any future vaccine. Moreover, a coronavirus strain is a patented by the Pirbright Institute, which is partially funded by the Bill and Melinda Gates Foundation.	https://www.usatoday.com/story/news/factcheck/2020/03/27/covid-19-fact-check-bill-melinda-gates-foundation-did-not-patent-coronavirus/2919503001/ (accessed on 1 December 2020)
Dr. Anthony Fauci is suspected to be a secret member of “deep state” which is attempting to undermine President Donald Trump. Besides, Conspiracy theorists claim Fauci has exaggerated the number of deaths from COVID-19 or accused him of “being a beneficiary to find treatments and a vaccine”.	https://www.moneycontrol.com/news/photos/coronavirus/miracle-cures-new-world-order-and-a-bill-gates-plan-the-most-absurd-coronavirus-conspiracy-theories-5930401-11.html (accessed on 1 December 2020)
Trump claims ‘deep state’ is delaying coronavirus vaccine until after US election.	https://www.independent.co.uk/news/world/americas/us-politics/trump-deep-state-coronavirus-vaccine-tweet-a9683406.html (accessed on 1 December 2020)
The imaginary “deep state”—a permanent, unelected shadow government which is said to have relentlessly gathered power to itself. some have extended it to include infectious-disease experts fighting the pandemic, such as Anthony Fauci.	https://www.newyorker.com/news/the-future-of-democracy/how-america-escapes-its-conspiracy-theory-crisis (accessed on 1 December 2020)
In September, the president tweeted unfounded allegations of the Food and Drug Administration in order to raise the suspicion that entrenched political of deep state forces were blocking the speedy testing and approval of a COVID-19 vaccine	https://newrepublic.com/article/159551/real-deep-state-trump (accessed on 1 December 2020)
Bill Gates’s plan is to reduce the world population by killing or control people through vaccinations.	https://www.aap.com.au/false-bill-gates-depopulate-with-vaccines-news-a-conspiracy-theory-classic/ (accessed on 1 December 2020)
The phone masts of 5G network are thought to contribute to the spread of disease.	https://theconversation.com/film-your-hospital-the-anatomy-of-a-covid-19-conspiracy-theory-147948 (accessed on 1 December 2020)
The COVID-19 pandemic is part of a strategy conceived by global elites—such as Bill Gates—to roll out vaccinations with tracking chips that would later be activated by 5G, the technology used by cellular networks.	https://www.npr.org/2020/07/10/889037310/anatomy-of-a-covid-19-conspiracy-theory (accessed on 1 December 2020)
The rollout of 5G has also been fraught with rumors and conspiracy theories—most recently, a narrative spread on social media that the wireless network technology fueled the coronavirus pandemic.	https://edition.cnn.com/2020/06/14/tech/5g-health-conspiracy-debunked/index.html (accessed on 1 December 2020)
A number of bot accounts, mixed with genuine accounts, made efforts to amplify the 5G conspiracy theory on social media. Some celebrities post suggestions on social media that 5G is linked to the spread of the virus or otherwise poses health risks.	https://tech.hindustantimes.com/tech/news/coronavirus-5g-conspiracy-theory-fuelled-by-coordinated-effort-story-mYiQfJ9UNNc6YKI6Mlj97L.html (accessed on 1 December 2020)
Twitter and Facebook timelines are being filled with people sharing their dreamed-up COVID-19 “cures”—like eating garlic soup, consuming silver-based supplements, drinking bleach, and even using cocaine.	https://nationalpost.com/news/no-cocaine-and-bleach-wont-cure-the-coronavirus-so-ignore-those-social-media-conspiracy-theories (accessed on 1 December 2020)
Conspiracy Theorists says a dangerous bleach cocktail solution can ‘cure’ the Wuhan coronavirus. Besides, a “miracle mineral solution”, a solution of 28 percent sodium chlorite in distilled water, was claimed useful by proponents.	https://highiqcommunity.com/conspiracy-theorists-say-a-dangerous-bleach-solution-can-cure-the-wuhan-coronavirus/ (accessed on 1 December 2020)
Colloidal silver is being touted and sold online as a killer of the novel coronavirus despite the lack of any evidence to support the claim.	https://disinformationindex.org/2020/04/covid-19-cures-colloidal-silver-and-the-original-health-hoax/ (accessed on 1 December 2020)
Colloidal silver was sold on the website as products to cure, mitigate, treat or prevent COVID-19, severe acute respiratory syndrome (SARS) and Middle East respiratory syndrome (MERS), among other diseases.	https://www.fda.gov/news-events/press-announcements/coronavirus-covid-19-update-federal-judge-enters-temporary-injunction-against-xephyr-llc-doing (accessed on 1 December 2020)
The video which is sponsored by the right-wing Tea Party Patriots shows a professional-looking group of people in white lab coats advocating hydroxychloroquine. One doctor speaking at the press conference promoted the drug as a “cure” for the coronavirus, and said that people “don’t need” to wear masks.	https://www.technologyreview.com/2020/07/28/1005738/hydroxychloroquine-covid-19-misinformation-video/ (accessed on 1 December 2020)
Miracle cures were by far the most prevalent conspiracy. President Trump’s advocacy for hydroxychloroquine and chloroquine as treatments/cures for COVID-19, though no peer-reviewed data found they had any efficacy in treating people suffering from the disease. Moreover, Trump claimed that ultraviolet light and disinfectants might be used to treat COVID-19.	https://www.forbes.com/sites/niallmccarthy/2020/10/05/the-most-common-coronavirus-conspiracies-circulating-in-the-media-infographic/?sh=bfee0bf4086d (accessed on 1 December 2020)
The COVID-19 pandemic has renewed the movement which advocates the bleach and the MMS	https://www.businessinsider.com/mms-bleach-advocates-exploiting-pandemic-sell-2020-7 (accessed on 1 December 2020)
President Trump has been a persistent and vocal advocate for the hydroxychloroquine. In April, he even declared: “What do you have to lose? Take it”. Besides, the drug has garnered a number of high-profile celebrities, including America’s pop queen Madonna, President Bolsonaro of Brazil, as well as Trump’s son, Donald Trump J. Some of them advocate it on the social media.	https://happymag.tv/hydroxychloroquine-the-conspiracy-theorists-answer-to-coronavirus-explained/ (accessed on 1 December 2020)
Some parties were held by somebody diagnosed by the COVID-19 virus. They wanted to see if the virus is real and to see if anyone gets infected.	https://www.the-sun.com/news/1122479/coronavirus-conspiracy-theorist-dies-virus-party/ (accessed on 1 December 2020)
A Florida mother allegedly took her high-risk teenage daughter to a church, tried treating the girl at home with hydroxychloroquine when she got sick, which led to her daughter’s death.	https://www.rawstory.com/2020/07/florida-teen-dies-after-conspiracy-theorist-mom-takes-her-to-church-covid-party-and-tries-to-treat-her-with-trump-approved-drug-report/ (accessed on 1 December 2020)
QAnon conspiracy theorists believe a deep state cabal of global elites is responsible for all the evil in the world. They also believe those same elites are seeking to bring down Trump, whom they see as the world’s only hope to defeat the deep state. QAnon has now brought the same conspiracy mentality to the coronavirus crisis.	https://theconversation.com/qanon-conspiracy-theories-about-the-coronavirus-pandemic-are-a-public-health-threat-135515 (accessed on 1 December 2020)
By virtue of his longtime public service, and his willingness to contradict the President, many of these conspiracy theorists see Fauci as a prime example of a Deep State advocate thwarting the President’s agenda.	https://www.forbes.com/sites/sethcohen/2020/07/14/is-fauci-a-deep-state-doctor/?sh=2982f20a7255 (accessed on 1 December 2020)
Not only the backers of are the QAnon conspiracy recommending drinking bleach as a cure against the coronavirus but also they have sought to link the spread of a the virus with a plot to depopulate the world hatched by Bill and Melinda Gates.	https://www.businessinsider.com/china-coronavirus-wuhan-cure-qanon-mms-bleach-conspiracy-2020-1 (accessed on 1 December 2020)
QAnon Followers believe the real reason Trump’s is in quarantine is to isolate him away from deep state plotters to arrest leftist deep-state operatives.	https://www.rollingstone.com/culture/culture-news/qanon-trump-coronavirus-conspiracy-theorists-1070131/ (accessed on 1 December 2020)
COVID-19 is a hoax designed to deflect attention from a Satan-worshipping pedophile ring operated by Hillary Clinton and liberal elites. Trump, their reasoning goes, is pretending to have COVID-19 as part of a grand plan to arrest Clinton.	https://www.theguardian.com/commentisfree/2020/oct/03/trump-coronavirus-conspiracy-theory-qanon (accessed on 1 December 2020)
President Donald Trump tweeted out a conspiracy theory that members of the “deep state”, had infiltrated Food and Drug Administration and were sabotaging efforts by pharmaceutical companies to enroll patients in coronavirus drug trials—all to stop him from winning reelection.	https://www.motherjones.com/coronavirus-updates/2020/08/trump-coronavirus-vaccine-deep-state/ (accessed on 1 December 2020)
The QAnon conspiracy theory has spread widely over recent months, migrating from far-right corners of the Internet. Its followers believe President Trump is a hero safeguarding the world from a “deep state” cabal of Satan-worshipping pedophiles, Democratic politicians and Hollywood celebrities.	https://time.com/5887437/conspiracy-theories-2020-election/ (accessed on 1 December 2020)
Death certificates have been knowingly manipulated by medical examiners to inflate the number of COVID-19 deaths	https://www.rollingstone.com/culture/culture-features/anti-vax-doctor-covid-19-death-certificates-984407/ (accessed on 1 December 2020)

**Table A2:** The states and district in the United States sorted according to their online attention values on the COVID-19-related conspiracy theories.

The States with Higher Attention on Conspiracy Theories	The States with Lower Attention on Conspiracy Theories
Alaska, Arizona, Arkansas, California, Colorado, Connecticut, Delaware, District of Columbia, Georgia, Hawaii, Idaho, Iowa, Illinois, Maine, Montana, Minnesota, Nebraska, Oklahoma, North Dakota, South Dakota, Tennessee, Texas, Utah, Vermont, Virginia, Wyoming	Alabama, Florida, Indiana, Kansas, Kentucky, Louisiana, Maryland, Massachusetts, Michigan, Missouri, Mississippi, Nevada, New Hampshire, New Jersey, New Mexico, New York, North Carolina, Ohio, Oregon, Pennsylvania, Rhode Island, South Carolina, Washington, West Virginia, Wisconsin
